# RNA-mediated pathogenic mechanisms in polyglutamine diseases and amyotrophic lateral sclerosis

**DOI:** 10.3389/fncel.2014.00431

**Published:** 2014-12-19

**Authors:** Ho Yin Edwin Chan

**Affiliations:** ^1^Laboratory of Drosophila Research, School of Life Sciences, Faculty of Science, The Chinese University of Hong KongHong Kong, China; ^2^Biochemistry Programme, School of Life Sciences, Faculty of Science, The Chinese University of Hong KongHong Kong, China

**Keywords:** polyglutamine disease, Drosophila models, C9orf72, repeat expansion-associated non-ATG translation, nucleolin

## Abstract

Gene transcription produces a wide variety of ribonucleic acid (RNA) species in eukaryotes. Individual types of RNA, such as messenger, structural and regulatory RNA, are known to play distinct roles in the cell. Recently, researchers have identified a large number of RNA-mediated toxicity pathways that play significant pathogenic roles in numerous human disorders. In this article, we describe various common RNA toxicity pathways, namely epigenetic gene silencing, nucleolar stress, nucleocytoplasmic transport, bi-directional gene transcription, repeat-associated non-ATG translation, RNA foci formation and cellular protein sequestration. We emphasize RNA toxicity mechanisms that involve nucleotide repeat expansion, such as those related to polyglutamine (polyQ) disorders and frontotemporal lobar degeneration-amyotrophic lateral sclerosis.

## Introduction

Ribonucleic acids (RNAs) are polymeric macromolecules composed of a wide variety of nucleotide building blocks. Various classes of RNA have been reported to date, of which major examples are messenger RNAs (mRNAs), ribosomal RNAs (rRNAs), transfer RNAs, and non-coding RNAs. Each RNA class is distinct in terms of synthesis, properties and functions. It is widely acknowledged that most RNAs have cellular roles in gene regulation and protein translation. However, a few RNAs are known to have less common functions such as serving as genetic materials for RNA viruses. In cells, RNAs are transcribed by RNA polymerase according to sequences on the DNA template. A subset of RNAs is further modified through various processing steps such as cleavage, base modification and editing. Such modifications are crucial to the generation of mature and fully functional RNA molecules.

**Figure 1:**
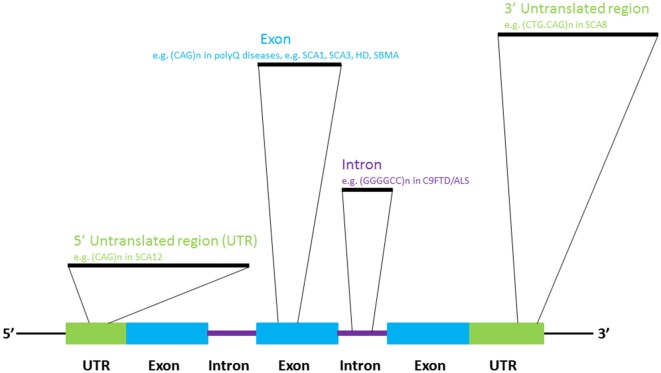
**Select diseases that display RNA repeat expansion-mediated toxicities**.

In addition to the well-established roles of various classes of RNA molecules in neuronal (Iyengar et al., 2014) and brain (Follert et al., 2014) development, many RNAs are known to be involved in neural pathologies (Cooper et al., 2009). Recent investigations have provided a more thorough understanding of the pathogenic roles of RNAs in neurological disorders. The dysregulation of the cellular processes that govern RNA metabolism is now known to contribute to neuronal dysfunctions and diseases. Such perturbation may be caused by the alteration of RNA transcription, splicing, editing and/or nuclear export due to genetic predisposition or as a consequence of normal aging (Cooper et al., 2009; Da Cruz and Cleveland, 2011; Johnson et al., 2012; Singh, 2012; Belzil et al., 2013b; Nalavade et al., 2013; Caillet-Boudin et al., 2014). Genome microsatellite instability, particularly nucleotide repeat expansion, has been shown to cause a number of human genetic diseases (Mirkin, 2007), many of which are neurological diseases such as amyotrophic lateral sclerosis (ALS), Huntington’s Disease (HD) and spinocerebellar ataxias (SCAs; Figure 1; Cruts et al., 2013; Nalavade et al., 2013; Tsoi and Chan, 2014). The toxic effect of nucleotide repeat expansion has been demonstrated to be caused by to a gain-of-function mechanism. Mutant RNA molecules that harbor expanded repeat sequences tend to form intracellular RNA foci, which are pathogenic hallmark of many of the diseases listed above (Wojciechowska and Krzyzosiak, 2011). The involvement of many RNA-binding proteins in human diseases is unsurprising, as RNAs often take the form of ribonucleoprotein complexes in cells (Lukong et al., 2008). Protein sequestration and RNA foci formation have been reported to play important roles in the gain-of-function pathogenic mechanisms that produce neuronal RNA toxicity. Mutant RNAs may also interfere with gene transcription via RNA-mediated gene silencing mechanisms (Colak et al., 2014). In addition, mutant RNAs may confer neurotoxicity at the protein level through a repeat-associated non-ATG translation mechanism (Cleary and Ranum, 2014). In this review, recent advances in research on the above mentioned RNA toxicity mechanisms will be discussed.

## Nucleotide repeat expansion as a pathogenic mechanism of neurodegeneration

In 1991, the mutations causing fragile-X syndrome (Kremer et al., 1991; Verkerk et al., 1991) and spinobulbar muscular atrophy/Kennedy’s disease (La Spada et al., 1991) were identified. Incidentally, the molecular pathogenic mechanisms of both diseases stem from the inter-generational expansion of genomic triplet nucleotide repeat sequences. To date, more than 20 neurological diseases have been reported to be caused by microsatellite sequence expansion mechanism (Polak et al., 2013), such as HD (Macdonald, 1993), several types of spinocerebellar ataxia (Rüb et al., 2013) and frontotemporal lobar degeneration-amyotrophic lateral sclerosis (DeJesus-Hernandez et al., 2011; Renton et al., 2011; Gijselinck et al., 2012; Figure 1). We now know that repeat-expansion mutation is closely associated with the unique structural features of repetitive DNA sequences, and perturbs cellular DNA replication, repair and recombination (Mirkin, 2007).

## Association between pathogenic pathways and CAG repeat expansion in the protein-coding region

The expansion of CAG trinucleotide repeats in the protein-coding regions of the human genome is the pathogenic mechanism for many neurological diseases (Nalavade et al., 2013). Protein translation leads to the production of proteins that harbor expanded stretches of glutamine amino acid residues. Various mouse models of polyglutamine (polyQ) diseases have been developed (Figiel et al., 2012) to examine the role of expanded polyQ proteins in disease pathogenesis. Since the beginning of the new millennium,* Drosophila* disease models have offered an alternative means of investigating the genetic pathogenic pathways involved in nucleotide-expansion diseases (McLeod et al., 2005), such as polyQ diseases (Chan and Bonini, 2000; Yu and Bonini, 2011). To investigate the pathogenic role of expanded CAG sequences at the RNA level, Li et al. (2008) expressed an artificial *DsRed* reporter that carried an expanded allele of CAG sequence located in the 3′ untranslated region of the transgene in the nervous system, and observed neurodegeneration in the retina and the brain. Intriguingly, toxicity was reduced when the CAG repeat continuity of the mutant allele was intermittently disrupted by a CAA codon. This indicates that the repeat continuity of CAG triplets is essential to RNA toxicity (Li et al., 2008). Expanded CAG RNA toxicity was also observed in *Caenorhabditis elegans* (Wang et al., 2011a) and mouse (Hsu et al., 2011) models.

## Expanded CAG RNA pathogenic mechanisms

### Alternative splicing of RNAs

**Figure 2:**
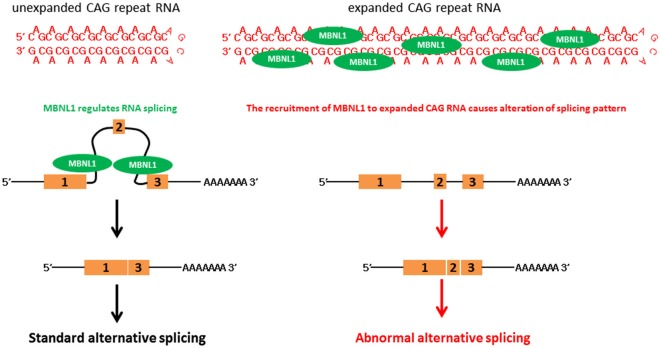
**CAG triplet repeat RNA causes aberrant RNA splicing**.

Expanded CAG RNAs form foci in cell and animal models, and in patient cells (Li et al., 2008; de Mezer et al., 2011; Hsu et al., 2011; Wang et al., 2011a; Wojciechowska and Krzyzosiak, 2011). Muscleblind-like (MBNL) proteins are a group of RNA-binding proteins that contain four zinc-finger domains, and are involved in the regulation of RNA alternative splicing (Konieczny et al., 2014). Artificial expanded CAG RNA binds to MBNL1 with a high affinity (~11 nM, as determined by filter-binding assay; Yuan et al., 2007). It was reported that MBNL1 protein sequestered to CAG RNA foci formed by *artificial CAG*, *ataxin-3* (*ATXN3*) and *huntingtin* (*htt*) transcripts (de Mezer et al., 2011; Hsu et al., 2011; Mykowska et al., 2011; Wang et al., 2011a). A group of MBNL1-regulated genes examined in cell models demonstrated alternative splicing alterations, such as neuronal cell lines that expressed expanded CAG constructs and SCA3 patient fibroblasts (Mykowska et al., 2011). The overexpression of MBNL1 may partially restore artificial expanded CAG RNA-induced alternative splicing defects in Mykowska et al. (2011). This demonstrates a correlation between MBNL1 recruitment to CAG RNA foci and MBNL1 dysfunction in the RNA toxicity of polyQ diseases (Figure 2). However, alternative splicing defects were not observed in an *in vivo DsRed-CAG*_270_ transgenic* Drosophila* model (Li et al., 2008). The overexpression of MBNL ortholog in a *C. elegans* expanded CAG RNA model may partially mitigate the expanded artificial CAG RNA phenotype of transgenic worms (Wang et al., 2011a).

### Toxicity of hairpin and double-stranded CAG repeat RNAs

**Figure 3:**
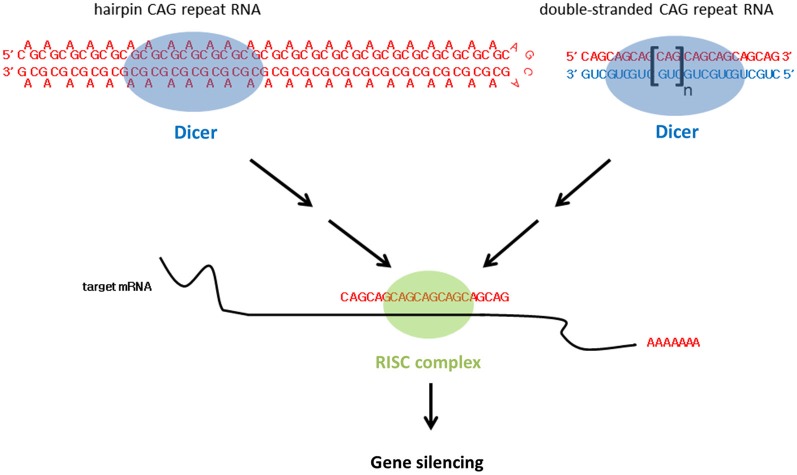
**CAG triplet repeat RNA leads to gene silencing**.

In addition to alternative splicing defects, small CAG RNAs are responsible for HD toxicity (Bañez-Coronel et al., 2012; Figure 3). Although these CAG RNA repeat sequences do not form foci in cells, they bind MBNL1 with an affinity similar to that of long CAG RNAs (Mykowska et al., 2011). Unexpanded short CAG repeat RNAs (sRNAs), such as (CAG)_7_ or (CAG)_20_, have been shown to induce alternative splicing defects in mammalian cells (Mykowska et al., 2011). More recently, Bañez-Coronel et al. (2012) detected *htt* small RNAs comprising approximately seven CAG repeats in cell models, mouse HD disease models and patient brain samples. The production of such sRNAs causes caspase activation and cytotoxicity and the number of CAG-repeat sRNAs is dependent on the CAG repeat length (Bañez-Coronel et al., 2012). Dicer is a RNase III family ribonuclease responsible for cleaving double-stranded precursor RNAs to generate sRNAs, and plays a key role in RNA interference (Bernstein et al., 2001). Expanded CAG RNAs form double-stranded RNA hairpins with A-A mismatches (Sobczak et al., 2003; Kiliszek et al., 2010; de Mezer et al., 2011; Busan and Weeks, 2013; Yildirim et al., 2013). Bañez-Coronel et al. (2012) showed that expanded CAG RNAs are good substrates of Dicer. A knockdown of Dicer expression reduces the level of CAG sRNAs in HD and SCA type 1 (Krol et al., 2007; Bañez-Coronel et al., 2012), which further indicates the involvement of RNAi machinery in the production of CAG sRNAs. Cleaved sRNAs participate in posttranscriptional gene silencing. The role of CAG sRNAs in RNA interference relies on their ability to interact with complementary CUG RNA tracts in cellular RNAs. The genes *MEIS2* and *ADORA2* have also been reported to be down-regulated in HD, and their transcripts have been predicted to form RNA duplexes with CAG sRNAs (Hodges et al., 2006). The overexpression of CAG sRNAs in cells reduces endogenous levels of* MEIS2* and *ADORA2* (Bañez-Coronel et al., 2012). This explains the role of CAG sRNAs in silencing cellular gene expression in CAG triplet repeat expansion diseases in general.

In addition to expanded CAG hairpin RNA, the transgenic expression of two complementary expanded CAG and CUG RNA sequences in the *Drosophila* eye causes deleterious external eye morphology (Lawlor et al., 2011; Yu et al., 2011). In contrast with CAG RNAs which adopt a double-stranded RNA hairpin structure with A-A mismatches (Sobczak et al., 2003; Kiliszek et al., 2010; de Mezer et al., 2011; Busan and Weeks, 2013; Yildirim et al., 2013), duplex CAG/CUG RNAs are expected to form perfectly-matched double-stranded CAG-CUG RNAs. In addition, Lawlor et al. showed that neither the expression of CAG nor CUG RNA elicits toxicity in flies (Lawlor et al., 2011), and that the rough eye phenotype induced by CAG-CUG co-expression was dependent on Dicer activity (Lawlor et al., 2011; Yu et al., 2011; Figure 3). sRNAs were reported in flies expressing single-stranded CAG hairpin RNAs and CAG-CUG duplex RNAs (Lawlor et al., 2011). However, CAG sRNAs were observed to be more prominent in the CAG-CUG dsRNA-expressing flies. At the molecular level, Lawlor et al. (2011) also detected altered miRNA levels in the CAG-CUG dsRNA-expressing flies. It is worth noting that miRNAs have been implicated in neurodegeneration (Abe and Bonini, 2013).

### Nucleolar stress and RNA toxicity

The results of a biochemical study conducted by our group indicate that in contrast with unexpanded CAG transcripts (both *ATXN3* and *artificial CAG* RNA), RNAs that carry expanded CAG repeats are enriched in the nuclear fraction (Tsoi et al., 2011). This finding suggests that nucleocytoplasmic transport of the mutant RNAs is perturbed. A follow-up genetic investigation using *Drosophila* model identified *U2AF50*, *Drosophila* ortholog of U2 small nucleoprotein auxiliary factor 65 (U2AF65; Blanchette et al., 2004), as a genetic modifier of expanded CAG RNA nucleocytoplasmic localization. The export of unexpanded *ATXN3* and pure CAG repeat transcripts was not affected. Knockdown of *U2AF50* expression in flies was found to intensify the neurodegenerative phenotype in flies (Tsoi et al., 2011). Tsoi et al. also identified a ribonucleoprotein complex consisting of mutant *ATXN3* RNA, U2AF65 and nuclear RNA export factor 1 in mammalian cells. This complex is responsible for mediating mutant RNA export. Knockdown of *U2AF65* expression in cells leads to the nuclear retention of mutant *ATXN3* RNA and triggers caspase activation (Tsoi et al., 2011). This finding confirms the involvement of the nucleus in expanded CAG RNA toxicity. Interestingly, the results of a HD mouse model study indicate that the level of developmental expression of U2AF65 decreases with age, and that this decline attenuates the nuclear export of mutant *htt* RNAs (Tsoi et al., 2011). This suggests that the nuclear accumulation of mutant *htt* RNA is a direct consequence of developmental decline in the cellular abundance of U2AF65, which coincides with the progressive degeneration of HD.

**Figure 4:**
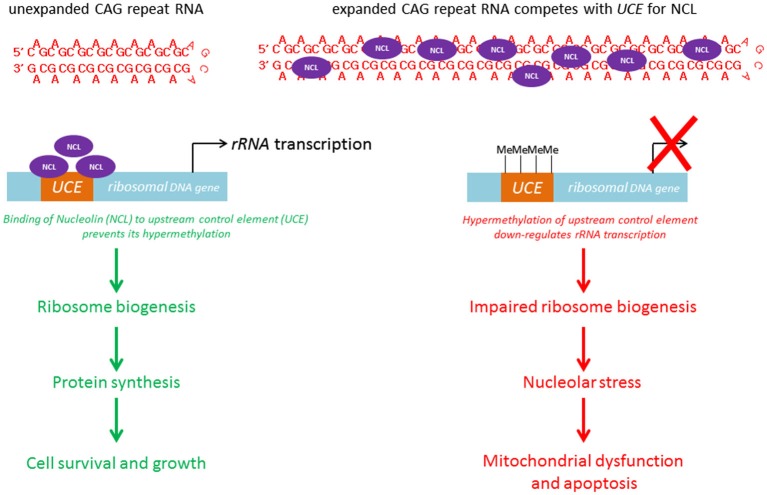
**CAG triplet repeat RNA induces nucleolar stress**.

Within the nuclear compartment, expanded CAG RNAs, including *ATXN3*, co-localize the nucleolar protein fibrillarin (Tsoi et al., 2012), suggesting that mutant RNAs interfere with nucleolar function (Figure 4). The cell nucleolus is the site of production of ribosome subunits, and the dysregulation of ribosome biogenesis has been shown to cause disease (Kressler et al., 2010). RNA polymerase I is responsible for the transcription of rRNA precursors (pre-rRNAs). rRNA is an essential component of the ribosome, a ribonucleoprotein complex responsible for protein translation. The inhibition of pre-rRNA transcription has been shown to cause apoptosis in neurons (Kalita et al., 2008). “Nucleolar stress” denotes the cellular pathway used by the nucleolus to communicate with cytosolic compartments (Boulon et al., 2010), such as the mitochondria (Lindenboim et al., 2011) and thereby initiate apoptosis. It is a universal and efficient mechanism for eliminating cells that are defective in protein synthesis due to ribosome biogenesis failure. Our group recently demonstrated a link between nucleolar stress and RNA toxicity in polyQ diseases, including SCA type 3 and HD (Tsoi et al., 2012; Tsoi and Chan, 2013, 2014). We found that nucleolin (NCL), a nucleolar protein that regulates rRNA transcription (Durut and Sáez-Vásquez, 2014), interacts directly and specifically with expanded CAG RNAs, and that such binding is mediated by the RNA-recognition motifs of NCL (Tsoi et al., 2012). This RNA/protein interaction prevents NCL protein from binding to the *upstream control element* (*UCE*) of the rRNA promoter, which results in *UCE* DNA hypermethylation. This leads to the down-regulation of rRNA transcription. We further showed that p53 protein becomes stabilized in cells and concentrated in the mitochondria. This causes the release of cytochrome c from the mitochondria, caspase activation and apoptosis induction (Tsoi et al., 2012). Although restoring the efficient nuclear export of mutant RNAs would alleviate nucleolar stress-mediated RNA toxicity (Tsoi and Chan, 2014), the exported cytosolic expanded CAG RNAs could cause the synthesis of expanded polyQ disease protein which might in turn trigger protein-mediated toxicity in cells (Williams and Paulson, 2008). To ensure a suitable therapeutic treatment for polyQ diseases, both RNA and protein toxicities must be effectively handled.

### Bi-directional transcription and repeat-associated non-ATG translation of CAG expansion transcripts

**Figure 5:**
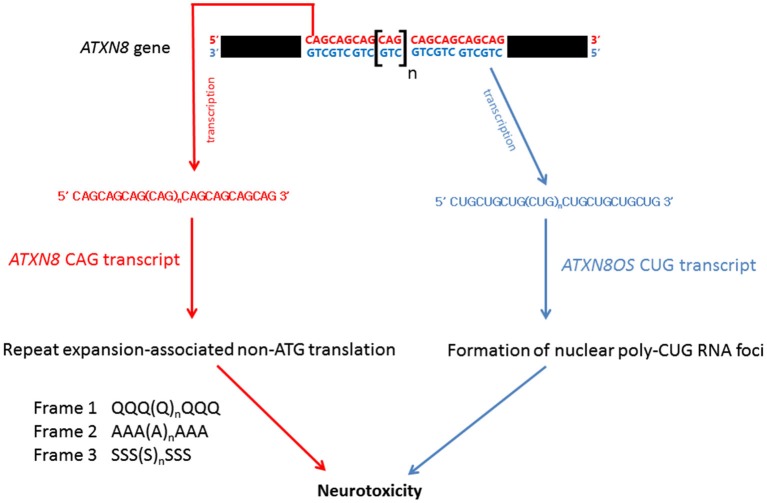
**RNA-mediated pathogenic pathways that associate with spinocerebellar ataxia type 8**.

Bi-directional transcription has been reported in the human genome, yielding both sense and anti-sense transcripts of complementary sequences. The anti-sense transcripts are now known to play important regulatory roles in eukaryotic gene expression (Faghihi and Wahlestedt, 2009). Spinocerebellar ataxia type 8 (SCA8) is caused by a CTG.CAG repeat expansion in the 3′ untranslated region of the *ataxin-8* gene (*ATXN8*; Figure 5; Koob et al., 1999). On gene transcription, the CAG-containing *ATXN8* and CUG-containing *ATXN8 opposite strand* (*ATXN8OS*) transcripts were detected in SCA8 patients (Moseley et al., 2006), and nuclear CUG *ATXN8OS* foci were found in HEK293 cells (Chen et al., 2009), the SCA8 mouse model and SCA8 patients (Daughters et al., 2009). Further, ribonuclear *ATXN8OS* foci were found to be co-localized with MBNL1 and displayed an alternative splicing pattern in both SCA8 mice and SCA8 patients (Daughters et al., 2009), demonstrating a gain-of-function role for expanded CUG *ATXN8OS RNA* in the pathogenesis of SCA8.

Under normal physiological conditions, protein synthesis is mediated by ATG initiation codon-dependent mRNA translation. Recently, Zu et al. (2011) discovered a repeat expansion-associated non-ATG translation (RAN) mechanism that facilitates protein synthesis in repeat expansion diseases. Interestingly, when an *ATXN8* transgene carrying a mutated ATG initiation codon was expressed in HEK293 cells, the CAG-containing region of the mutant *ATXN8* CAG transcripts was still able to produce protein products carrying a polyQ, a polyalanine or a polyserine domain (Zu et al., 2011). These homopolymeric proteins were translated from all three different reading frames of the ATG-defective *ATXN8* transcripts through the RAN mechanism (Figure 5). Long CAG repeat-containing transcripts are prone to frameshift error during protein translation and have been reported to cause the production of polyalanine and polyserine proteins in HD mouse model and HD patients (Davies and Rubinsztein, 2006), and polyalanine proteins in SCA3 patients (Gaspar et al., 2000). Both *in vitro* and *in vivo* analyses showed that these homopolymeric proteins are toxic and thus play a role in pathogenesis (Zu et al., 2011; van Eyk et al., 2012).

### Non-translating expanded CAG repeat sequences up-regulate gene transcription

Spinocerebellar ataxia type 12 is caused by the expansion of CAG triplet sequences in exon 7 of the brain-specific regulatory subunit of the protein phosphatase 2A gene *PPP2R2B* which constitutes part of the non-coding region of the gene (Holmes et al., 1999). The *PPP2R2B* promoter, which carries an expanded CAG allele, demonstrated greater transcriptional activity (Lin et al., 2010), and the transgenic overexpression of the human *PPP2R2B* gene in *Drosophila* caused neurodegeneration (Wang et al., 2011b), indicating that, in addition to glutamine-coding CAG triplet expansion, non-translating CAG repeat sequences can cause toxicity via the up-regulation of gene expression.

### Double-stranded RNA-dependent protein kinase binds mutant *htt* transcripts and its activity is induced in HD

Protein kinase R (PKR), also known as double-stranded RNA-dependent protein kinase, regulates protein translation by mediating the protein phosphorylation of eukaryotic initiation factor 2α (eIF2α; Marchal et al., 2014). Double-stranded RNAs, such as viral RNAs, have been shown to be the main activators of PKR. Expanded CAG transcripts, including *htt* and the *androgen receptor* (for spinobulbar muscular atrophy) RNAs, were reported to form double-stranded hairpin structures (de Mezer et al., 2011; Busan and Weeks, 2013). When *in vitro* transcribed biotinylated mutant *htt* RNA was incubated with human brain extracts, PKR was identified as the *htt* RNA-binding protein (Peel et al., 2001), demonstrating that *htt* transcripts are able to interact with PKR. The autophosphorylation of PKR is an indication of its activation (Marchal et al., 2014), and phospho-PKR immunoreactivity was detected in a HD transgenic mouse model and HD patients (Peel et al., 2001; Bando et al., 2005). As PKR activation causes the phosphorylation of eIF2α and subsequently leads to the induction of apoptosis (Peel, 2004), the binding of mutant *htt* transcripts to PKR may serve as a trigger to initiate neuronal cell death in HD.

### Other pathways

The results of recent genome-wide microarray and genetic analyses (Shieh and Bonini, 2011; van Eyk et al., 2011) have shown that in addition to alternative splicing (Mykowska et al., 2011), mRNA down-regulation (Bañez-Coronel et al., 2012), miRNA alteration (Lawlor et al., 2011) and nucleolar stress (Tsoi et al., 2012; Tsoi and Chan, 2013), other gene pathways are responsible for expanded CAG RNA toxicity. However, detailed mechanisms of these pathways are yet to be fully elucidated.

## Association between pathogenic pathways and GGGGCC repeat expansion in non protein-coding regions

**Figure 6:**
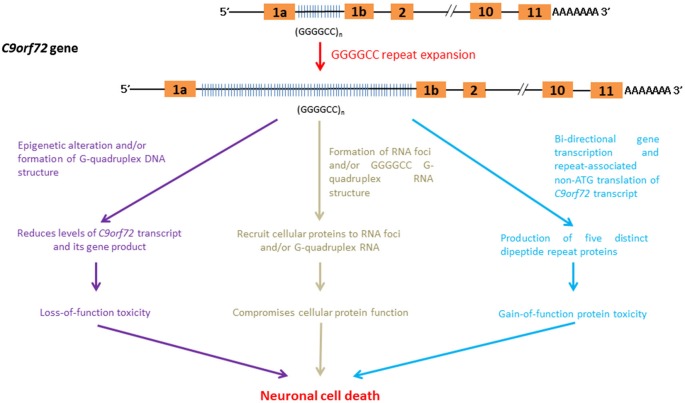
**RNA-mediated pathogenic pathways that associate with GGGGCC hexanucleotide repeat expansion in C9FTD/ALS**.

The expansion of non-coding GGGGCC repeats in a gene named *Chromosome 9 open reading frame 72 (C9orf72)* has been identified as a cause of ~40% of hereditary ALS and 25% of familial frontotemporal dementia (FTD) cases. Together, these conditions are generally termed C9FTD/ALS (DeJesus-Hernandez et al., 2011; Renton et al., 2011). Recent findings indicate that gain-of-function toxicity contributes significantly to C9FTD/ALS pathogenesis. Here, we discuss some recent advances in RNA-mediated gain-of-function toxicity in C9FTD/ALS (Figure 6).

### Influence of epigenetic regulation and G-quadruplex DNA formation on *C9orf72* gene expression

The *C9orf72* gene product belongs to the guanine nucleotide exchange factor protein family and has been implicated in intra-cellular membrane trafficking (Zhang et al., 2012; Levine et al., 2013). The down-regulation of *C9orf72* gene expression leads to toxicity due to a loss-of *C9orf72* function in C9FTD/ALS. The GGGGCC-repeat region lies between two alternatively spliced non-coding exons of the *C9orf72* gene (DeJesus-Hernandez et al., 2011; Belzil et al., 2013a). The findings of various independent studies indicate that the expression level of *C9orf72* transcripts is reduced in C9FTD/ALS patients (DeJesus-Hernandez et al., 2011; Gijselinck et al., 2012; Belzil et al., 2013a; Fratta et al., 2013). Recently, Waite et al. (2014) detected a reduction in C9ORF72 protein levels in C9FTD/ALS patients with the GGGGCC repeat expansion. This finding further supports the hypothesis that C9FTD/ALS entails a loss-of-protein function.

Post-translation modifications of the core histone proteins, H3 and H4, are known to modulate gene regulation. Trimethylation of H3 and H4 core histones at particular lysine residues are associated with gene silencing (Barski et al., 2007). Belzil et al. (2013a) showed that the gene expression level of *C9orf72* was reduced in patients who presented with H3K9, H3K27, H3K79 and H4K20 trimethylation. In addition to histone modifications, the intrinsic biophysical properties of *C9orf72* GGGGCC hexanucleotide repeat sequences also help to reduce the gene expression of *C9or72*. The appearance of four consecutive guanine nucleotides in DNA, such as in the case of GGGGCC in *C9orf72*, leads to the formation of a meta-stable DNA secondary structure termed a G-quadruplex (Haeusler et al., 2014). The formation of G-quadruplex DNA has been shown to play regulatory roles in various cellular processes, such as gene transcription control (Lam et al., 2013). Haeusler et al. (2014) recently demonstrated that the formation of GGGGCC DNA G-quadruplex structure halts the transcription of *C9orf72*, which provides an alternative explanation for the down-regulation of *C9orf72* gene expression observed in C9FTD/ALS.

### *C9orf72* RNA foci and cellular protein sequestration

*C9orf72* RNA containing GGGGCC repeats has been reported to form RNA foci (DeJesus-Hernandez et al., 2011; Almeida et al., 2013; Donnelly et al., 2013; Gendron et al., 2013; Lagier-Tourenne et al., 2013; Mizielinska et al., 2013; Sareen et al., 2013; Zu et al., 2013). Mizielinska et al. (2013) also identified an inverse correlation between RNA foci and age-at-onset of C9FTD/ALS. Biochemical and microscopic investigations showed that *C9orf72* RNA is capable of recruiting cellular proteins to RNA foci (reviewed by Vatovec et al., 2014). Among these proteins are RNA-binding proteins such as hnRNPs (Lee et al., 2013; Mori et al., 2013b; Sareen et al., 2013) and RNA export factors (Sareen et al., 2013; Cooper-Knock et al., 2014). The recruitment of these proteins to *C9orf72* RNA foci compromises their cellular functions, leading to C9FTD/ALS pathologies.

### Recruitment of cellular components to *C9orf72* DNA and RNA G-quadruplexes

G-quadruplex structures (Haeusler et al., 2014) have been detected in DNAs and RNAs (Kikin et al., 2008). Recently, GGGGCC repeat sequences have been found to promote the formation of *C9orf72* RNA G-quadruplex (Fratta et al., 2012), whose structure is sequence- and GGGGCC repeat length-dependent (Reddy et al., 2013). Haeusler et al. (2014) identified a series of cellular proteins that interact specifically with GGGGCC RNA G-quadruplexes (Haeusler et al., 2014). The direct interaction of nucleolar protein NCL with GGGGCC RNA G-quadruplexes was confirmed (Durut and Sáez-Vásquez, 2014). More importantly, the subcellular localization of NCL was found to be altered in patient cells, and NCL was shown to become more diffusely localized outside the nucleolar region in cells expressing expanded GGGGCC *C9orf72* RNA (Haeusler et al., 2014). The subcellular mislocalization of NCL also affects pre-rRNA processing in patient cells. This finding is indicative of nucleolar stress activation (Boulon et al., 2010), and provides a direct molecular link between *C9orf72* GGGGCC RNA G-quadruplexes and C9FTD/ALS toxicity. In addition to NCL, GGGGCC G-quadruplex DNA and RNA structures have also recently been reported to possess heme-binding activity (Grigg et al., 2014). Heme is composed of ferrous iron and protoporphyrin, and serves as a prosthetic group of many cellular proteins, such as redox enzymes. Its role in neurodegeneration is well illustrated in Alzheimer’s Disease. Amyloid beta peptide has been found to bind with heme (Atamna and Frey, 2004), leading to heme deficiency (Atamna et al., 2001). It is obvious that the recruitment of cellular components to GGGGCC G-quadruplex structures triggers neurotoxicity in C9FTD/ALS via various pathogenic pathways.

### Bi-directional transcription and repeat-associated non-ATG translation of *C9orf72*

Although GGGGCC repeat expansion is located in non-coding exon of *C9orf72* (DeJesus-Hernandez et al., 2011; Belzil et al., 2013a), Mori et al. (2013a) showed that GGGGCC repeats are transcribed bi-directionally. More recently, both the sense and anti-sense GGGGCC *C9orf72* transcripts have been reported to generate five different dipeptide repeat (DPR)-containing proteins composed of GA, GP, GR, PR and AP amino acid repeats (Ash et al., 2013; Gendron et al., 2013; Mori et al., 2013a,c; Zu et al., 2013) via the RAN mechanism (Zu et al., 2011). Microscopic DPR protein aggregates have been detected in C9FTD/ALS patients (Liu et al., 2014; May et al., 2014; Proudfoot et al., 2014). It has been shown that DPR protein aggregates are heterogeneous in nature, and co-localize with cellular proteins such as the proteasome degradation marker p62 (Mackenzie et al., 2013; Mann et al., 2013; Mori et al., 2013a,c; May et al., 2014), ubiquitin (Zhang et al., 2014) and transport factor Unc119 (May et al., 2014). The co-localization of cellular proteins to DPR aggregates is expected to cause varying degrees of loss-of-function in the cellular proteins, which in turn contributes to C9FTD/ALS pathogenesis.

The neurotoxicity of DPR proteins is determined by their subcellular localization and by the sequestration of cellular proteins to DPR aggregates. Kwon et al. (2014) demonstrated that the nucleolar localization of DPR aggregates impairs pre-rRNA biogenesis and causes cell death. The results from an independent study conducted by Zhang et al. (2014) indicate that cytosolic DPR aggregates impair the ubiquitin-proteasome system (UPS) and induce ER stress. As both the UPS and ER stress pathways are involved in protein homeostasis, these findings highlight the role of protein misfolding in C9FTD/ALS. In addition to cell culture models, *Drosophila* models have been used to demonstrate the neurotoxicity of DPR polypeptides (Mizielinska et al., 2014). In other words, DPR protein toxicity has been confirmed using an *in vivo* animal disease model.

## Therapeutic development to combat nucleotide repeat expansion RNA toxicity

Based on our current understanding of nucleotide repeat expansion disease pathogenesis pathways, various therapeutic approaches have been developed. Oligonucleotide-based therapeutics (Fiszer and Krzyzosiak, 2014) such as antisense oligonucleotides (ASOs) have been shown to reduce *C9or72* RNA expression and foci formation (Donnelly et al., 2013; Lagier-Tourenne et al., 2013; Sareen et al., 2013), and thereby to reduce RNA toxicity in C9FTD/ALS. Small molecules capable of targeting toxic RNA-protein interaction can pharmacologically correct splicing defects associated with sequestration of MBNL1 in expanded CAG RNA (Kumar et al., 2012), and hnRNPA1 in expanded GGGGCC *C9orf72* RNA (Zamiri et al., 2014). In addition, another class of small molecules that target GGGGCC RNAs has been reported to be capable of reducing RNA foci formation and RAN-mediated DPR protein production (Su et al., 2014) in C9FTD/ALS. Prior to clinical trials, animal disease models will be used to test these novel compounds and approaches. It is expected that the identification of more nucleotide repeat expansion pathogenic pathways will enable more therapeutic approaches to be developed in the future.

## Outlook

Although protein toxicity was considered to play a major role in the pathogenesis of repeat expansion diseases, we now know that mutant transcripts also induce cell dysfunction and death via multiple mechanisms, such as the alteration of gene expression levels and splicing patterns, the generation of small RNAs, the induction of nucleolar stress, the promotion of bi-directional transcription and repeat-associated non-ATG translation of the disease locus, the activation of apoptotic signaling, and the sequestration of cellular components to RNA foci. The above RNA-mediated mechanisms are predicted to operate in conjunction with the protein-mediated pathways to confer overall neurotoxicity. We now know that mutant RNAs confer cytotoxicity by sequestering cellular components to RNA foci (Wojciechowska and Krzyzosiak, 2011). The identification of the individual polyQ protein species, including the monomer, oligomer, protofibril, fibril and inclusion body, has greatly facilitated the study of polyQ protein toxicity (Hands and Wyttenbach, 2010). Technologies and strategies that result in a detailed classification of individual mutant RNA species will allow more in-depth and systematic investigations of the RNA-mediated pathogenesis of neurodegeneration.

## Conflict of interest statement

The author declares that the research was conducted in the absence of any commercial or financial relationships that could be construed as a potential conflict of interest.
